# To Remove Burnt Powder from the Skin

**Published:** 1900-11

**Authors:** A. Everett Smith

**Affiliations:** Olean, N.Y., 107 South Barry Street


### Clinical Reports.

### TO REMOVE BURNT POWDER FROM THE SKIN.

By A. EVERETT SMITH, B. S., M.D., Olean, N.Y.

PHYSICIANS who have had the experience of slowly picking powder burned and driven into the face by accidental or premature explosion will appreciate the following method by which it can be removed rapidly and completely in a few minutes. I do not recall reading of any one having used this method and it was only the force of circumstances by which I accidentally discovered it.

July 3, 1899, I was called to see C. S., aged about 7 years whose face was burned and filled with powder by the explosion of a giant fire-cracker. I found my colleague, Dr. M. C. Follett, of this city, at the bedside, who stated that the child's eyes were probably ruined and his face would be permanently disfigured, since it would be simply impossible to pick all the powder out. Dr. Follett having an urgent call elsewhere left me to attend to the child's eyes. I soon discovered it was impossible to do anything without general anesthesia and requested the family to have Dr. Follett meet me an hour later. It was then late in the day and darkness came on before we could meet, so we deemed it best to wait until morning.

The next morning or some fifteen hours after the accident, we placed the child under chloroform anesthesia, and I proceeded to wash the face preparatory to the picking job, I thought was before me. Soap and water, with a coarse towel took off the blackened outside layer of the skin loosened by the burn and inflammatory process, when to my surprise I found it quite easy to wash and rub away the powder from the tissue thus exposed. Afterward with an oily dressing to exclude the air, the case made an uninterrupted recovery with scarcely a black mark in his skin.

My conclusions are in all similar cases, best results will be attained by leaving patient for ten or twelve hours until the serum from the inflammatory process forms, then give a general anesthesia and scrub the parts thoroughly with soap and water.

107 South Barry Street.

